# Subclinical hypothyroidism and depression: a meta-analysis

**DOI:** 10.1038/s41398-018-0283-7

**Published:** 2018-10-30

**Authors:** T. Zhao, B. M. Chen, X. M. Zhao, Z. Y. Shan

**Affiliations:** 1 Department of Endocrinology and Metabolism, Institute of Endocrinology, First Affiliated Hospital, China Medical University, Shenyang, Liaoning China; 2 First Affiliated Hospital of Sun Yat-sen University, Department of Hepatobiliary Surgery, Guangzhou, China; 3 Chengde Medical University, Chengde, Hebei China

## Abstract

The objective of this study was to evaluate the relationship between subclinical hypothyroidism (SCH) and depression. We also analysed the effect of levothyroxine (L-T4) on depression in SCH patients. We found an insignificant difference for the composite endpoint: standard mean difference (SMD) of 0.23 (95% confidence interval (CI) −0.03, 0.48, *P* = 0.08, *I*^2^ = 73.6%). The odds ratio (OR) for depressive patients was 1.75 (95% CI 0.97, 3.17 *P* = 0.064, *I*^2^ = 64.6%). Furthermore, sub-group analysis according to age found that SCH was related to depression in younger patients (<60 years old), as defined by the diagnosis of depression: OR of 3.8 (95% CI 1.02, 14.18, *P* = 0.047, *I*^2^ = 0.0%) or an increase on the depressive scale: SMD of 0.42 (95% CI 0.03, 0.82, *P* = 0.036, *I*^2^ = 66.6%). Meanwhile, SCH did not associate with depression in older patients (≥60 years old), as defined by the diagnosis of depression: OR of 1.53 (95% CI 0.81, 2.90, *P* = 0.193, *I*^2^ = 71.3%) or an increase on the depressive scale: SMD of 0.03 (95%CI −0.31, 0.37, *P* = 0.857, *I*^2^ = 79.8%). We also found an insignificant difference in the composite endpoint between the L-T4 supplementation group and placebo group in SCH patients. The estimated SMD was 0.26 (95% CI −0.09, 0.62, *P* = 0.143, *I*^2^ = 52.9%). This meta-analysis demonstrates that SCH is not connected to depression. However, sub-group analysis according to age found that SCH is related to depression in younger patients, but not in older patients. Furthermore, we failed to find an effect of L-T4 supplementation treatment for SCH on depression.

## Introduction

The connection among overt thyroid dysfunction, abnormal mood, and cognitive disorder has been well documented^1,2^. Overt hypothyroidism is a cause of major mood disorder, including melancholia, and may result in deteriorating dementia^1^. Recently, several reports have focused on subclinical hypothyroidism (SCH) and its latent neuropsychiatric and neurocognitive outcomes; however, these studies have failed to determine the precise nature and strength of this relationship. SCH or “mild hypothyroidism” is characterised by elevated thyroid-stimulating hormone (TSH) levels with normal free circulating thyroid hormones (FT4) and is a usual dysfunction of the thyroid axis^3^. SCH may impact up to 17.6 percent of patients in the general population.

Several reports have demonstrated a connection between SCH and current depressive symptoms, current major depression, and a lifetime history of major depression. Meanwhile, other papers have failed to detect this association. Moreover, the relationship between depressive symptoms and SCH is controversial, particularly in older patients, as the prevalence of SCH increases with age. SCH may impact up to 22% of women >60 years old and is slightly less prevalent in men^4,5^.

In cases of overt thyroid disorder, treating psychiatric symptoms with L-T4 supplementation is well indicated. However, few randomised controlled trials have investigated L-T4 supplementation for the treatment of SCH^6,7^, and the evidence regarding its effect on depressive symptoms is limited.

We performed a meta-analysis of the available evidence from cross-sectional reports and both prospective and retrospective studies investigating the relationship between SCH and depressive scale. In addition, we examined the association between SCH and depression and the effect of L-T4 replacement on depression in SCH patients.

## Subjects and methods

### Search strategy

We searched the PubMed, EMBASE, MDELINE, and COCHRANE databases for studies in all languages published by June 2017 in all languages. We selected papers officially published in English. The studies were identified and evaluated separately by two authors (Zhao and Chen) by searching for the major medical subject headings “hypothyroidism” and “depression” in the subsequent text and key words. The following search formula is an example of the strategy used to search the MEDLINE database: ((“hypothyroidism”[MeSH Terms] OR “hypothyroidism”[Text Word]) AND ((Asymptomatic [Text Word] OR mild [Text Word]) OR subclinical [Text Word])) AND ((((((“depressive disorder”[MeSH Terms] OR “depression”[MeSH Terms] OR Depression[Text Word]) OR (“depressive disorder”[MeSH Terms] OR Depressive Disorder[Text Word])) OR (“adjustment disorders”[MeSH Terms] OR Adjustment Disorders[Text Word])) OR (“mood disorders”[MeSH Terms] OR Affective Disorders[Text Word])) OR (“bipolar disorder”[MeSH Terms] OR Bipolar Disorder[Text Word])) OR (“depressive disorder, major”[MeSH Terms] OR “depressive disorder”[MeSH Terms] OR Major Depressive Disorder[Text Word])). To avoid missing any relevant papers, we also reviewed the reference lists of key reports for related articles.

### Study selection

Two reviewers (Zhao and Chen) separately evaluated all potential titles and abstracts. Articles were precluded if the title and/or abstract were not suitable for the meta-analysis. Subsequently, we collected the full versions of the qualified reports. Disagreements were settled by consensus and the view of a third author (Zhao) as necessary. SCH is characterised by elevated levels of TSH and normal fT4 levels^8^. However, the upper limit of the TSH reference range is controversial. Several experts propose that the TSH upper limit is 4.5–5.0 mIU/L^8,9^, but other authors recommend for the upper limit to be lowered to 2.5–3.0 mIU/L due to the increased risk of developing overt hypothyroidism and a higher prevalence of anti-thyroid antibodies compared to that among euthyroid participants^9^. Due to this controversy, a particular TSH upper limit for the SCH definition was not pre-identified in this meta-analysis. In addition, the fT4 values were considered regular if they were perceived as normal by the reviewers, even though details regarding the fT4 values were not indicated. The papers were considered qualified if they met the corresponding criteria: published data, cross-sectional study, case control report, prospective study, retrospective study, RCTs, clearance and use of a unique commercial product to perform the TSH assay, specified depressive measurement and domains, and number of depressed patients included (with well-described criteria for depression). Reviews, case reports, letters, nonhuman studies, and conference abstracts were eliminated. Studies involving subjects who were formerly diagnosed with major depressive disorder, neurological diseases such as stroke or Parkinson’s or severe medical conditions that could influence mood, such as cancer, studies neglecting to mention the scales used to evaluate depression and studies in which most patients with SCH were treated with L-T4 were excluded from the study.

### Data extraction and quality assessment

Two authors (Zhao and Chen) independently extracted the data from the original studies using a uniform standard procedure. Dissent was resolved by consensus and the recommendation of a third observer (Zhao) as required. We recorded information including the publication year, first name of the authors, type of study, SCH definition, age, depression evaluation (prevalence or incidence of depression), Hamilton Rating Scale for Depression (HDRS), Hospital Anxiety and Depression Scale (HAD), Beck Depression Inventory (BDI), Geriatric Depression Scale (GDS), Psychological General Well-Being Index (PGWI), Center for Epidemiologic Studies-Depression Scale (CES-D), subject gender, and sample size.

The quality of the included reports was evaluated using the Newcastle-Ottawa Scale (NOS)^10^. The NOS includes eight items regarding selection, comparability, exposure and consequence. The scale is scored from zero to nine stars, and the highest score indicates the best methodological quality.

The methodological quality of the selected RCTs was evaluated according to the Cochrane Handbook for Systematic Review of Interventions based on the following aspects: generation of random sequence, distribution concealment, blinding, incomplete consequence date description, free of selective reporting, and free of other bias.

### Statistical analysis

First, because not all studies used identical units of measurement, we quantified the outcomes as the standard mean difference (SMD) with the 95% confidence interval (CI) for successive variables. Then, the results were calculated as the odds ratio (OR) with 95% CI for the discrete variables. Statistical heterogeneity across the original studies was estimated by performing a Cochrane’s *Q*-test (*P* < 0.05 indicated statistical significance) and the *I*^2^ test (*I*^2^ > 50%: high heterogeneity; *I*^2^ = 25–50%: medium heterogeneity; and *I*^2^ < 25%: low heterogeneity)^11^. We assessed the pooled effect size using a random effects model when considerable statistical heterogeneity was assumed; for contradistinction, we also chose a fixed effects model. *P* < 0.05 was considered a statistically significant difference. We performed pre-specified subgroup analyses to evaluate the between-thesis heterogeneity according to age (≥60 or <60 years).

For the RCTs, statistical analysis was also performed to determine the efficacy of L-T4 on depression in SCH patients. We performed statistical analyses using STATA 11 for Windows.

### Sensitivity analysis and risk of bias

To determine the impact of the individual studies on the summary risk assessment, we performed a one-study-removed analysis by omitting one report from each rotation and re-computing the pooled estimates of the remaining studies employing the metaninf command.

Publication bias was investigated in the meta-analysis by performing a funnel plot and Begg’s test. The funnel plot is a straightforward approach of investigating whether publication bias is present. Furthermore, we used Begg’s test to examine the symmetries of the plots statistically.

## Results

### Study selection

In total, 261 articles were identified by the preliminary search of articles published by June 10, 2017; of these articles, 157 were more comprehensively evaluated and 17 papers were finally selected for the meta-analysis (Supplementary Figures 1).

### Study characteristics

Table 1 illustrates the characteristics of the five cross-sectional and nine prospective studies included in the meta-analysis. In total, the 14 studies involved 5678 participants, including 715 participants diagnosed with SCH. The selected papers defined SCH by the upper reference limit of TSH (the TSH cut-off value), which ranged from 4.0 to 5.6 mIU/L. Eight of the 14 studies also reported fT4 measurements. The studies used various depressive tests to assess a wide scope of depressive domains. The quality of the included papers was moderate or good, and the NOS scores ranged from five to seven (Table 1). Table 2 indicates the characteristics of the RCTs included in the meta-analysis.

**Table 1:** The characteristics of non-RCTs

First author	Publication year	Article type	*N* Total	N SCH	Cut-off TSH mIU/l	fT4 reference pmol/l	Age	Gender	Depression test	Study quality scale
Selcuk	2005	Cross-sectional	22	7	N/A	N/A	42.5	Man/Woman	HDRS; BSI; HAD	7
Robin	2007	Cross-sectional	320	80	4.0	10·0–19·0	54.3	Woman	PGWI	6
Robert	2016	Cross-sectional	35	17	4.5	N/A	30.0	Woman	BDI	7
Seref	2006	Prospective	63	43	5.5	8.9–18.0	42.7	Man/Woman	HDRS	8
Manuel	2015	Prospective	593	47	4.5	12.0–18.0	75.0	Man/Woman	GDS	6
Marina	1997	Prospective	36	19	4.6	6.3–15.3	52.9	Woman	HDRS	6
Young	2010	Prospective	918	164	4.1	7.0–18.0	76.8	Man/Woman	GDS	6
Renate	2011	Prospective	1185	64	4.5	N/A	74.5	Man/Woman	CES-D	7
Manciet	1995	Prospective	393	26	4.5	N/A	65*	Man/Woman	CES-D	6
Ajay	2014	Cross-sectional	1591	141	N/A	10.1–17.9	70*	Man/Woman	BDI	6
Silvana	2014	Cross-sectional	278	43	4.0	8.0–19.0	80.4	Man/Woman	GDS	6
Rolf	2006	Prospective	186	38	5.0	N/A	61.3	Man/Woman	BDI	7
Robert	2017	Prospective	24	12	4.5	N/A	34.5	Man	BDI	5
Gokhan	2010	Prospective	34	14	5.6	7.5–21.2	38.9	Woman	BDI	7

**Table 2:** The characteristics of RCTs

First author	Year	Country	N	TSH cut-off mIU/l	fT4 reference pmol/l	Depression test	Intervention
Parle	2010	UK	94	5.5	9.0–20.0	HADS	Initial dosage being 25 ug of L-T_4_ or placebo per day
Vaneska	2012	Brazil	57	4.0	11.6–23.2	BDI	Initial dosage of L-T4 based on TSH values from 25 µg to 75 µg or placebo per day
Laily	2015	Iran	60	4.5	10.3–25.8	BDI	Received 100 µg of L-T_4_ (Iran Hormone Product) or placebo per day
Rolf	2006	Norway	69	4.2	9–22	BDI	Dosage of L-T4 based on TSH values from 25 µg to 175 µg or placebo per day

The quality of the RCT studies was good (Supplementary Figures 2).

### Outcomes

**Fig. 1:**
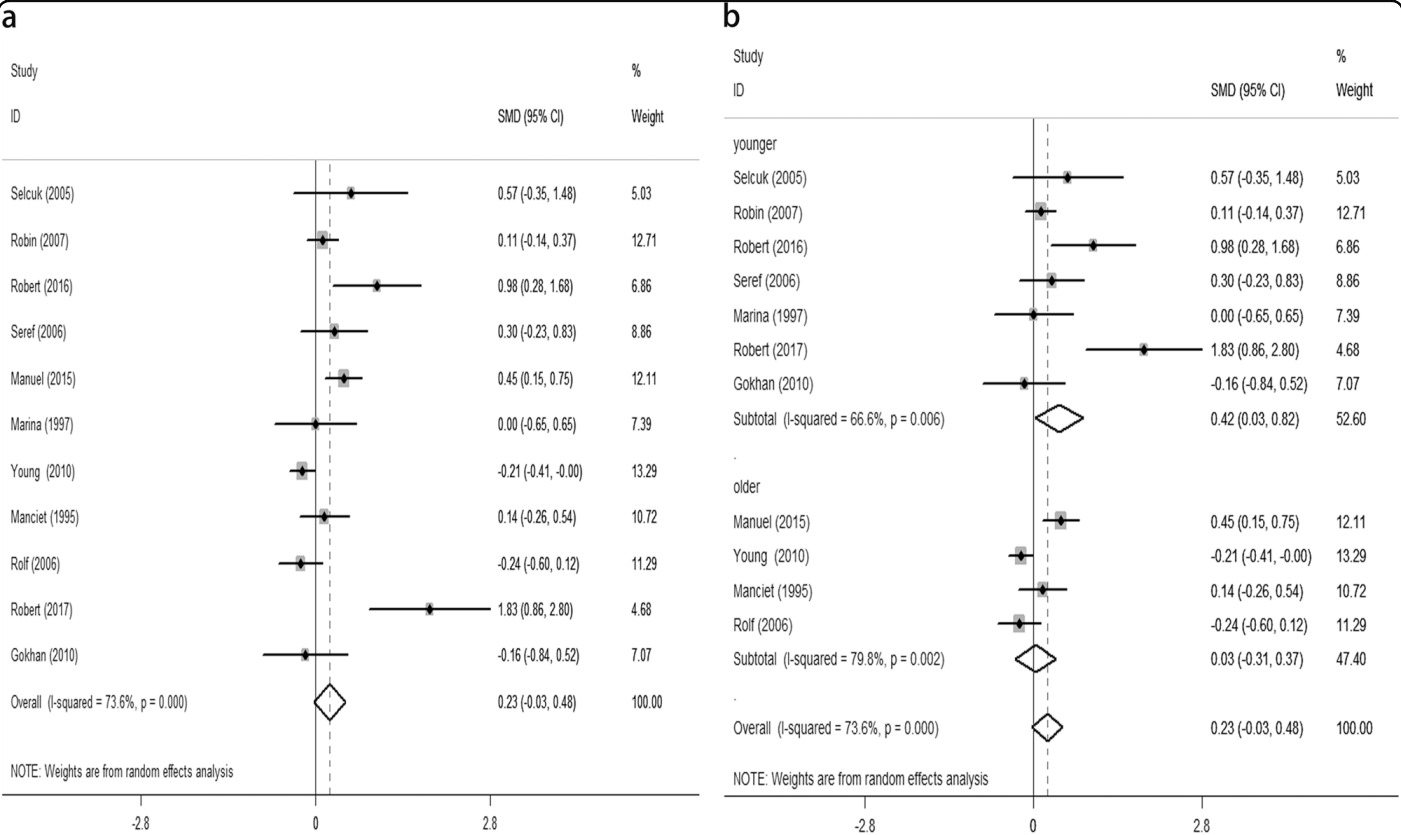
**a** Forest plots showing standard mean differences (SMD, 95% CI) for the increase of depressive score in SCH patients comparing to the normal individuals in a random effects model. X-axis: possitive values equal to the aggravation on depressive tendency. **b** Subgroup analyses of depressive scale based on age in a random effects model. X-axis: possitive values equal to the aggravation on depressive tendency. SMD: standard mean differences. Younger: participants with the mean age <60 years old. Older: participants with the mean or minimum age ≥60 years old

**Fig. 2:**
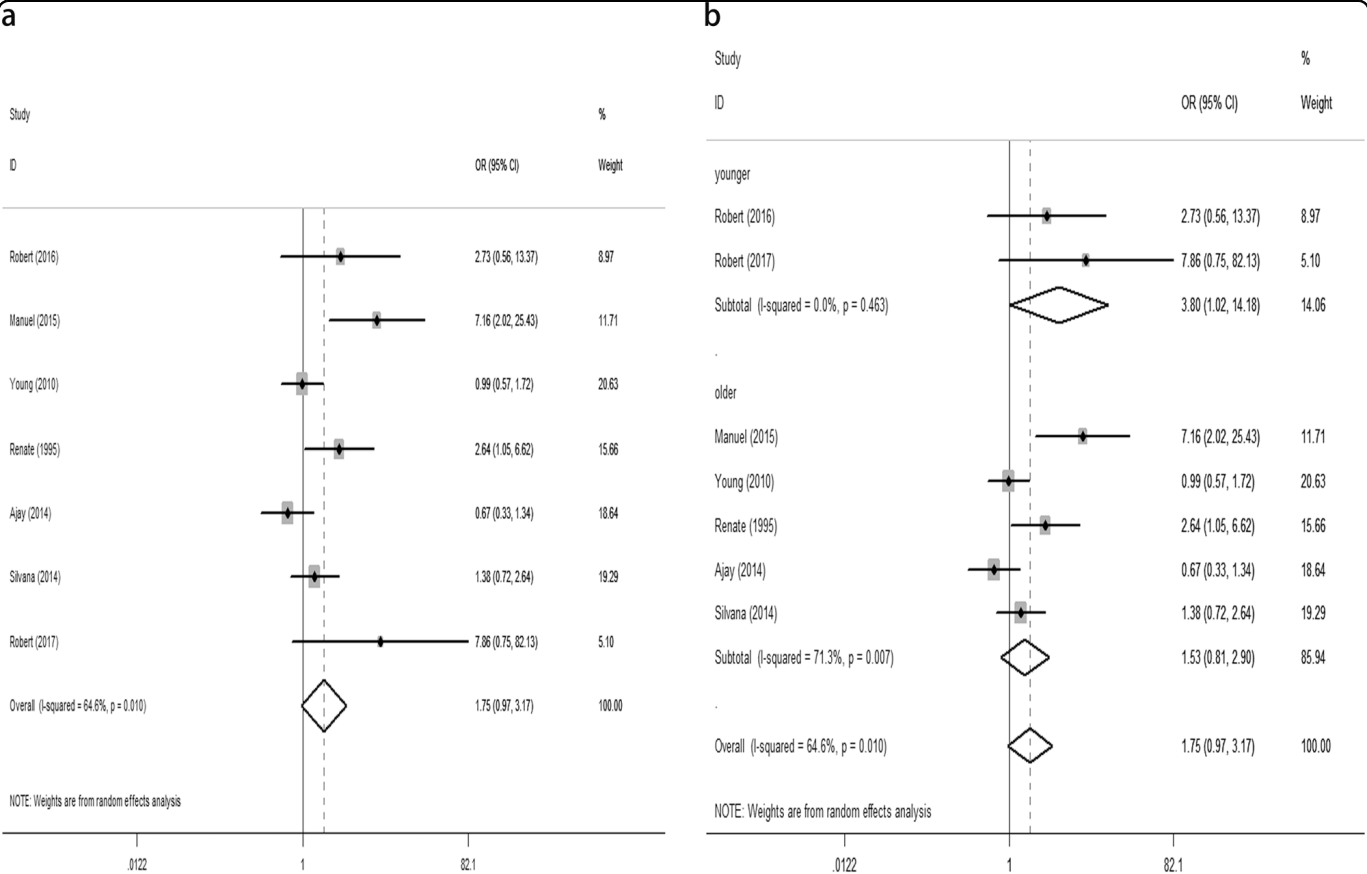
**a** Forest plots of studies comparing the number of depression patients between SCH and euthyroid individuals. The rhombus represents the OR and 95% CI obtained for the combined calculation. **b** Subgroup analyses of depression based on age in a random effects model. The rhombus represents the OR and 95% CI obtained for the combined calculation. Younger: participants with the mean age <60 years old. Older: participants with the mean or minimum age ≥60 years old

**Fig. 3:**
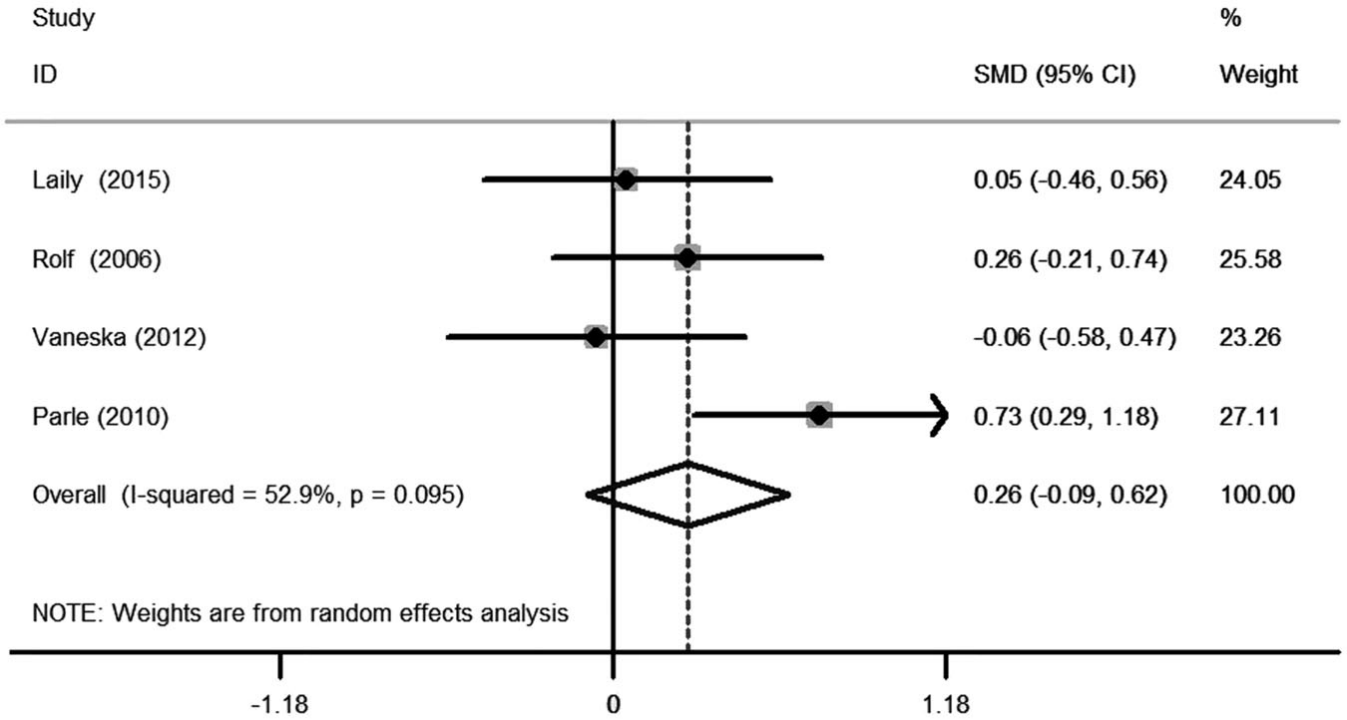
Forest plots showing standard mean differences (SMD, 95% CI) for improvement in depressive scale comparing L-T4 treatment to the placebo group in a random effects model. X-axis: positive values equal to the aggravation on depressive tendency

Overall, we found an insignificant difference in the following composite end point: the SMD for depressive scores was 0.23 (95% CI −0.03, 0.48, *P* = 0.08, *I*^2^ = 73.6%) (Fig. 1a). The OR for depressive patients was 1.75 (95% CI 0.97, 3.17, *P* = 0.064, *I*^2^ = 64.6%) (Fig. 2a). However, the sub-group analysis by age demonstrated that SCH was associated with depression in the younger patients (<60 years old) but not in the older patients (≥60 years old) (Figs. 1b, 2b). No significant difference was observed in the composite end point between the L-T4 therapy group and placebo group in SCH patients (Fig. 3). The estimated SMD was 0.26 (95% CI −0.09, 0.62, *P* = 0.143, *I*^2^ = 52.9%).

### Publication bias and sensitivity analysis

We performed sensitivity analyses by sequentially eliminating one study at a time to probe the change in the total SMD and 95% CI of the depressive domain. The sensitivity bias analysis of these original articles revealed no variation in the orientation of the OR when each publication was omitted (Supplementary Figure 3). In addition, using the SMD as the endpoints, no significant effect was observed (Supplementary Figure 4). Publication bias was assessed by performing Begger’s linear regression analysis of both the composite outcome and risk of depression (*P* = 0.230 and *P* = 0.087, respectively) (Supplementary Figures 5 and 6). The consequences of the Begg’s test suggested no significant publication bias was existed.

## Discussion

Overall, this meta-analysis indicates that mild thyroid dysfunction is related to depression in younger patients (<60 years old) as determined by the diagnosis of depression or an increase on the depressive scale. We found an insignificant connection between depression and SCH in the older patients (≥60 years old). Furthermore, we failed to detect an influence of L-T4 supplementation therapy for SCH on depression.

Numerous studies have investigated the mechanism by which overt thyroid disease influences physical, behavioural, and cognitive functions^9^; however, the connection between SCH and these consequent measures has not been rigorously defined, and studies have reported contradictory outcomes^12–16^.

In recent years, many publications have paid increasing attention to the relationship between SCH and depression; however, these studies revealed tremendous heterogeneity in the recruited population, particularly in terms of age, gender, and depressive domains. Notably, articles involving participants varying in age may affect mutual comparability. In recent decades, numerous studies have investigated the relationship between age and depression. Many studies have reported that age is an inconvenient element that influences the path and symptomatology of a depressive episode^17,18^. Depressive episodes accompanied by anxiety and the risk of committing suicide are considerable predictors that differ according to the age of the subjects. We performed a sub-group analysis because of the clinical heterogeneity. SCH was found to be associated with depression in the younger adults (<60 years old). The only difference between SCH and normal thyroid function is TSH. Two large publications have reported an indistinguishable link with lower T4 and/or higher TSH levels in subjects presenting with depressive syndrome (based on the depression region of the Diagnostic Interview Schedule) among 6869 younger subjects from the United States^19^ and worse psychological well-being (based on self-reported questionnaires) among 2269 younger men from the United Kingdom^20^. Several studies using animal models have examined the relationship between SCH and depression, which was demonstrated by increased immobility performance on the forced swimming test (FST) and tail suspension test (TST)^21,22^. In 2014, an animal study illustrated that the density of hippocampal T3 was slightly lower in the SCH group than the sham group, which indicated a dysfunction in thyroid hormones in the brains of SCH rats even though their plasma thyroid hormones were in the normal range^21^. Many papers have investigated whether SCH promotes depression-like behaviour in rats in conjunction with slight hyper-activity of the hypothalamus-pituitary-adrenal (HPA) axis. Furthermore, several subsequent studies have investigated the role of the Wnt signalling pathway in mood diseases, such as major depression^23^ and bipolar disorder^24^. Thyroid hormones trigger cell proliferation, restrain the delivery of key elements of the Wnt signalling pathway and suppress β-catenin levels^25^. Consequently, the Wnt/β-catenin pathway appears to participate in SCH-related depression.

Recently, many publications have investigated whether SCH is connected to a higher risk of cognitive damage and depression in older populations^26–29^. However, the results are conflicting, and epidemiological articles investigating this linkage have reported inconstant results. In 2015, two meta-analyses failed to provide evidence supporting the association between SCH and cognitive impairment in older subjects^30,31^. We also observed no relationship between SCH and depression in older patients. A probable interpretation of this disparity could be the over-diagnosis of SCH among the elderly due to the lack of age-associated serum TSH reference ranges in the original studies included in the meta-analysis^32^. Indeed, movement of the serum TSH distribution curve to higher levels during ordinary ageing may result in misclassification of older patients as suffering from SCH^33,34^. Based on the National Health and Nutrition Examination Survey study, the 97.5th percentile of the standard serum TSH concentration is 3.9 mIU/L in adults younger than 49 years and 6.3 mIU/L in adults older than 80 years^34^. Thus, the younger individuals likely had “true” SCH at the same serum TSH levels, while the older adults (>80 years) did not, which could have resulted in an underestimation of the association between the risk of depression and SCH among the elderly. Another reasonable interpretation is that many older patients with biochemical outcomes consistent with SCH would have reverted to a normal thyroid function state without L-T4 replacement. Stott et al. have demonstrated that three-fifths of older people screened for enrolment into the trial based on formerly increasing thyroid hormone values had returned to normal thyroid biochemical levels without L-T4 treatment^35^. Many of the included articles did not re-examine the thyroid hormone levels over time. Using this approach, avoiding the inclusion of several people with normal thyroid hormone into the group of SCH patients is challenging, which could result in an underestimation of the risk of depression associated with SCH among the elderly.

Another meaningful consequence of the current meta-analysis is demonstrated by the ineffectiveness of therapy for SCH with LT4 supplementation on depression. Recently, a paper from New England found no significant differences in the mean alteration in the Tiredness score or Hypothyroid Symptoms score over a 1-year period between a placebo group and an L-T4 treatment group among older people with SCH. This finding is similar to our results. Furthermore, several non-placebo controlled trials have demonstrated improvements in depressive scores with L-T4 replacement in SCH patients^36,37^. Based on these outcomes, it seems difficult to believe that L-T4 treatment truly improves depressive symptoms in SCH patients; this conclusion is supported by the similar outcomes in the L-T4 treatment and placebo groups. These similar results suggest that patients are particularly susceptible to the efficacy of being treated regardless of the treatment (the “placebo effect”). However, in animal studies, thyroid hormones affect the neurotransmission of serotonin and noradrenaline, which play critical roles in the pathogenesis of depression and are the targets of current antidepressant therapy^38–41^. These publications reveal elevated serotonin levels in the cerebral cortex of rats after T3 administration. Furthermore, lower serotonin levels are observed in brains with hypothyroidism. Serotonin exerts a repressive effect on thyrotropin-releasing hormone (TRH) excretion, suggesting the presence of a feedback loop that permits the activation of the hypothalamus-pituitary-thyroid axis if serotonin values in the brain are relatively low. Overall, the current evidence does not demonstrate a significant benefit of L-T4 treatment. High-quality placebo-controlled studies with large sample sizes are needed.

### Strengths and limitations of this meta-analysis

This analysis has four main limitations. First, some data were obtained from observational studies, many of which are cross-sectional studies. Second, probable bias existed in the selection of the included studies, including quality problems in the original articles, inevitable heterogeneity, publication bias, and confounding factors. To restrict bias in the selection of the included studies, moderate inclusion criteria were utilised to identify studies that produced measurable data related to the risk of depression in participants with SCH. Third, possible bias may have existed due to the heterogeneity of the meta-analysed studies in terms of SCH definition (based on the upper limit of TSH) and in the depressive evaluations (diagnosis of depression and depressive scales). Fourth, none of the selected reports applied age-adjusted expected limits, resulting in the unavoidable misclassification of older adults as suffering from SCH. In addition, the diagnosis of SCH was typically based on a unitary assessment of TSH without at least a second verified measurement. This method can lead to the potential misclassification of normal thyroid hormone participants who only have a transient elevation in the serum TSH level.

Despite these limitations, the current meta-analysis has enhanced the statistical power by pooling the results of the studies. Thus, the overall number of cases and controls was adequately large to support the conclusion of the meta-analysis. Additionally, we evaluated the benefit of L-T4 administration for depression in SCH patients based on RCTs. We used precise methodology. Consistent with the distinct original data, we chose different effect models. Consistent with the diverse original reports, we applied different methods of evaluating the quality.

## Conclusions

Overall, this meta-analysis indicates that SCH is not linked to depression, which was defined by a diagnosis of depression or an increase on the depressive scale. In the younger patients (<60 years old), SCH was associated with depression. However, in the older patients (≥60), SCH was not related to depression. Furthermore, we failed to find any benefit of L-T4 treatment on depression in SCH patients. Further studies using multiple age-associated TSH reference ranges can provide a better evaluation of the risk of depression in SCH patients.

## Electronic supplementary material

Supplementary Figure 1

Supplementary Figure 2

Supplementary Figure 5

Supplementary Figure 6

Supplementary Figure 3

Supplementary Figure 4

Supplemental legends
