# Supplementary material for: Subclinical hypothyroidism and depression: a meta-analysis
Source: Transl Psychiatry. 2018 Oct 30;8:239. doi: 10.1038/s41398-018-0283-7 (PMC6207556; doi:10.1038/s41398-018-0283-7)
Supplement: Supplementary file 1 — Supplementary Figure 1 [file 41398_2018_283_MOESM1_ESM.pdf]

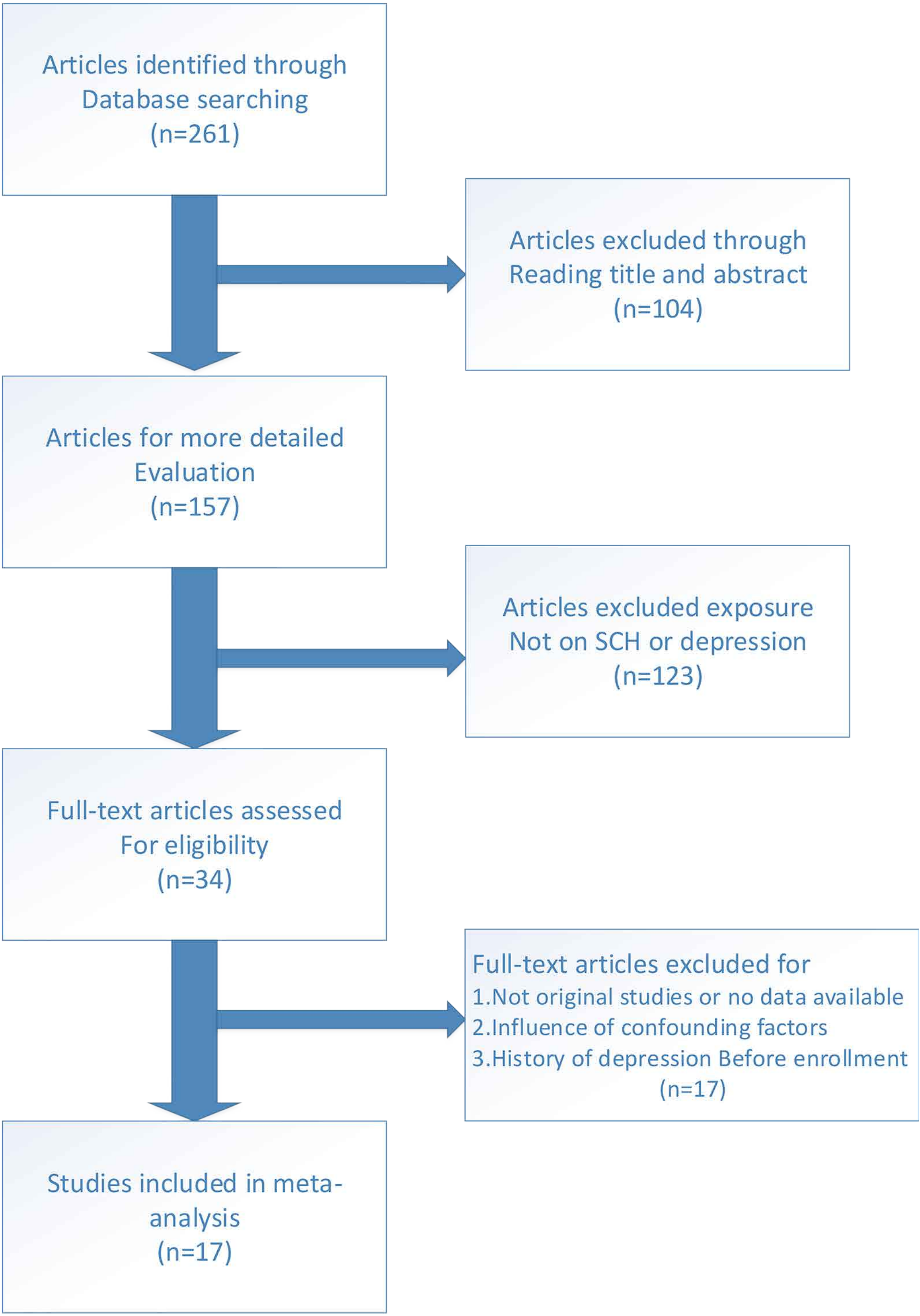
Articles identified through Database searching (n=261)
Articles excluded through Reading title and abstract (n=104)
Articles for more detailed Evaluation (n=157)
Articles excluded exposure Not on SCH or depression (n=123)
Full-text articles assessed For eligibility (n=34)
Full-text articles excluded for
1.Not original studies or no data available
2.Influence of confounding factors
3.History of depression Before enrollment
(n=17)
Studies included in meta-analysis (n=17)
