# Supplementary material for: Subclinical hypothyroidism and depression: a meta-analysis
Source: Transl Psychiatry. 2018 Oct 30;8:239. doi: 10.1038/s41398-018-0283-7 (PMC6207556; doi:10.1038/s41398-018-0283-7)

	Random sequence generation (selection bias)	Allocation concealment (selection bias)	Blinding of participants and personnel (performance bias)	Blinding of outcome assessment (detection bias)	Incomplete outcome data (attrition bias)	Selective reporting (reporting bias)	Other bias
J. Parle 2010	-	+	+	+	+	+	?
Laily Najafi 2015	?	+	+	+	+	+	+
Rolf Jorde 2006	?	+	+	-	+	+	?
Vaneska Spinelli Reuters 2012	-	+	+	+	+	+	?

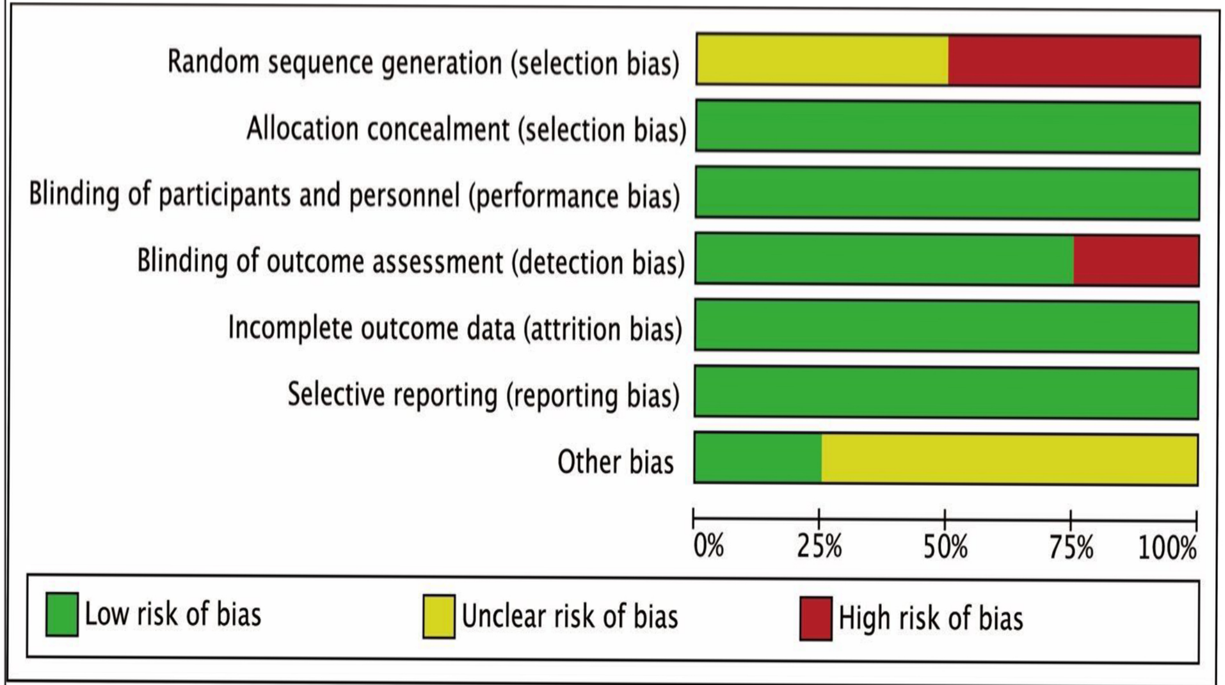

Supplement: Supplementary file 2 — Supplementary Figure 2 [file 41398_2018_283_MOESM2_ESM.pdf]
