# Supplementary figures and images for: Subclinical hypothyroidism and depression: a meta-analysis
Source: Transl Psychiatry. 2018 Oct 30;8:239. doi: 10.1038/s41398-018-0283-7 (PMC6207556; doi:10.1038/s41398-018-0283-7)

Begg's funnel plot with pseudo 95% confidence limits

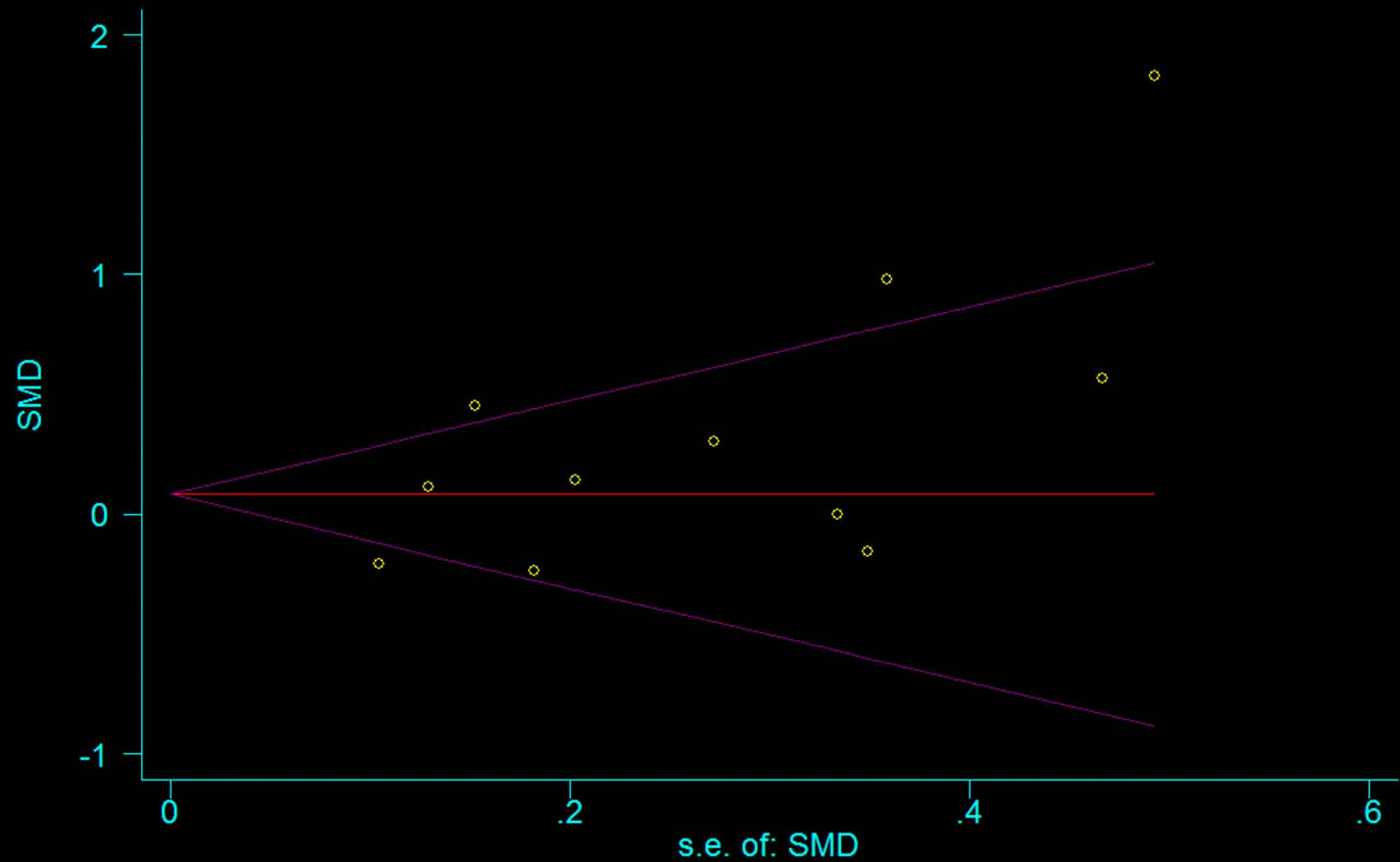

Supplement: Supplementary file 3 — Supplementary Figure 5 [file 41398_2018_283_MOESM3_ESM.pdf]

Begg's funnel plot with pseudo 95% confidence limits

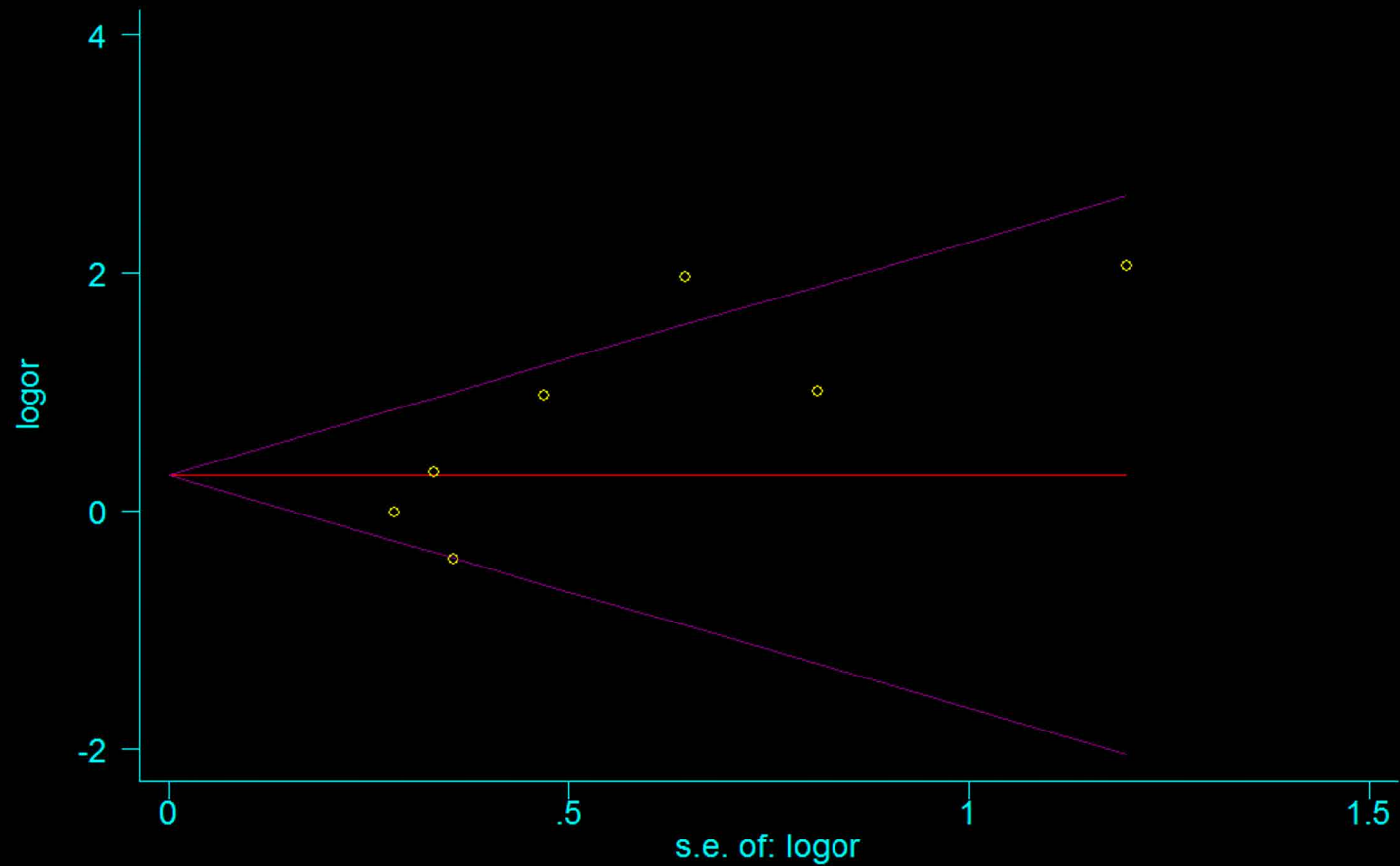

Supplement: Supplementary file 4 — Supplementary Figure 6 [file 41398_2018_283_MOESM4_ESM.pdf]

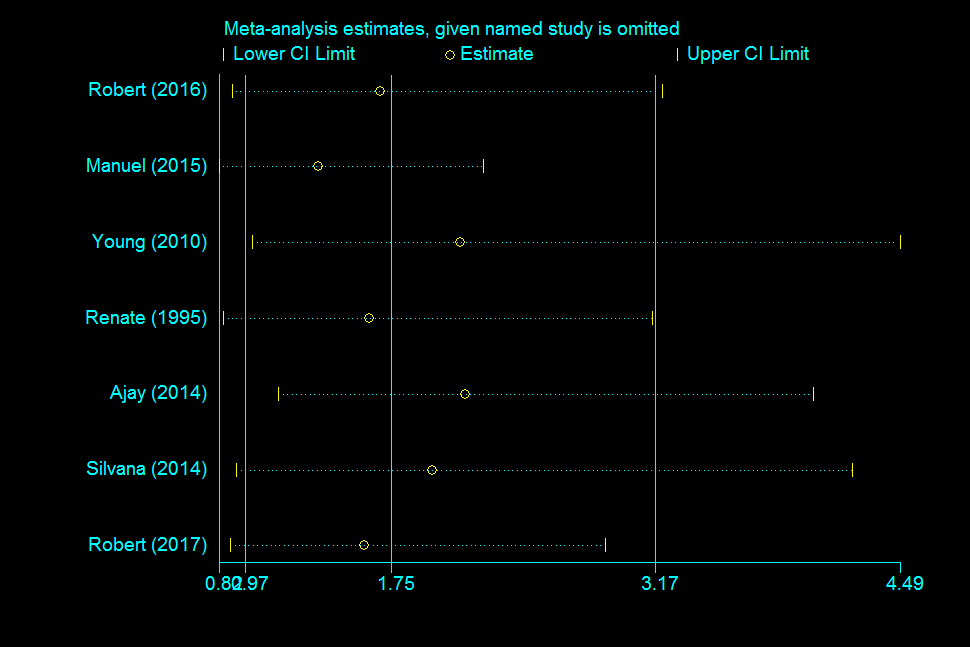

Supplement: Supplementary file 5 — Supplementary Figure 3 [file 41398_2018_283_MOESM5_ESM.tif]

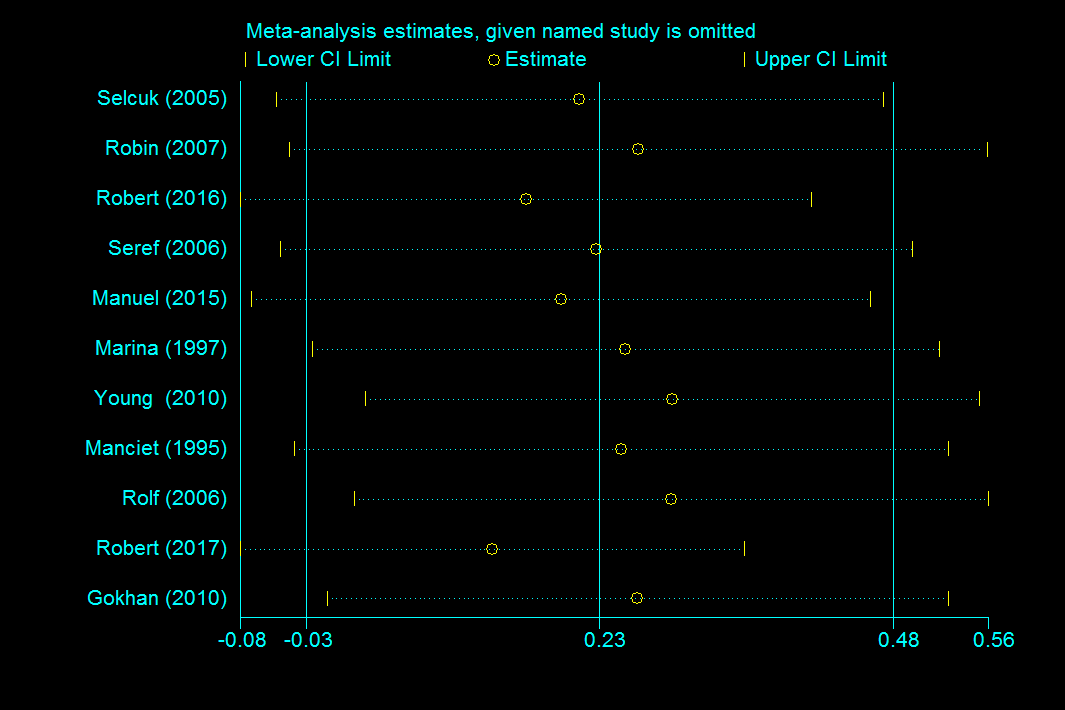

Supplement: Supplementary file 6 — Supplementary Figure 4 [file 41398_2018_283_MOESM6_ESM.tif]
