# Supplementary material for: Subclinical hypothyroidism and depression: a meta-analysis
Source: Transl Psychiatry. 2018 Oct 30;8:239. doi: 10.1038/s41398-018-0283-7 (PMC6207556; doi:10.1038/s41398-018-0283-7)
Supplement: Supplementary file 7 — Supplemental legends [file 41398_2018_283_MOESM7_ESM.docx]

Supplementary Figure 1. Process of study selection. SCH, subclinical hypothyroidism.

Supplementary Figure 2. Quality assessment of the RCTs.

Figure 1a. Forest plots showing standard mean differences (SMD, 95% CI) for the increase of depressive score in SCH patients comparing to the normal individuals in a random effects model. X-axis: possitive values equal to the aggravation on depressive tendency.

Figure 2a. Forest plots of studies comparing the number of depression patients between SCH and euthyroid individuals. The rhombus represents the OR and 95% CI obtained for the combined calculation.

Figure 1b. Subgroup analyses of depressive scale based on age in a random effects model. X-axis: possitive values equal to the aggravation on depressive tendency. SMD: standard mean differences. Younger: participants with the mean age <60 years old. Older: participants with the mean or minimum age≥60 years old.

Figure 2b. Subgroup analyses of depression based on age in a random effects model. The rhombus represents the OR and 95% CI obtained for the combined calculation. Younger: participants with the mean age <60 years old. Older: participants with the mean or minimum age≥60 years old.

Figure 3. Forest plots showing standard mean differences (SMD, 95% CI) for improvement in depressive scale comparing L-T4 treatment to the placebo group in a random effects model. X-axis: positive values equal to the aggravation on depressive tendency.

Supplementary Figure 3. Sensitivity analysis of the studies included in the meta-analysis. The figure shows the OR obtained by combined analysis of the remaining studies after the successive exclusion of each study individually. The excluded study is listed on the left, and the corresponding horizontal lines indicate the OR and CI obtained by re-calculation after its exclusion. The CI for the overall meta-analysis of the studies is indicated by two vertical lines.

Supplementary Figure 4. Sensitivity analysis of the studies included in the meta-analysis. The figure shows the SMD obtained by combined analysis of the remaining studies after the successive exclusion of each study individually. The excluded study is listed on the left, and the corresponding horizontal lines indicate the SMD and CI obtained by re-calculation after its exclusion. The CI for the overall meta-analysis of the studies is indicated by two vertical lines.

Supplementary Figure 5. Begg’s funnel plot for publication bias analysis. Each point represents a separate study.

Supplementary Figure 6. Begg’s funnel plot for publication bias analysis. Each point represents a separate study.
